# A Morphology-Guided Conditional Generative Adversarial Network for Rapid Prediction of Hazard Gas Dispersion Field in Complex Urban Environments

**DOI:** 10.3390/s26082367

**Published:** 2026-04-11

**Authors:** Zeyu Li, Suzhen Li

**Affiliations:** 1 College of Civil Engineering, Tongji University, Siping 1239, Shanghai 200092, China; 2032403@tongji.edu.cn; 2 State Key Laboratory of Disaster Reduction in Civil Engineering, College of Civil Engineering, Tongji University, Siping 1239, Shanghai 200092, China

**Keywords:** hazard gas dispersion, conditional generative adversarial networks, morphology analysis, Lattice Boltzmann method, gas sensing and detection, real-time forward modeling

## Abstract

The accurate and rapid prediction of hazard gas dispersion fields in urban environments is essential for guiding emergency sensor deployment and enabling real-time risk assessment. However, the computational cost associated with Computational Fluid Dynamics (CFD) simulations hinders their use as real-time forward models, while simplified Gaussian plume models lack the fidelity to resolve building obstruction effects. This study proposes a morphology-guided conditional Generative Adversarial Network (cGAN) framework designed to achieve real-time gas dispersion field modeling in urban environments with complex building configurations. The urban area is discretized into 50 × 50 m grid cells, each characterized by six morphological parameters describing building geometry. K-means clustering categorizes these cells into distinct morphological types. High-fidelity dispersion datasets are then generated for each type using Lattice Boltzmann Method (LBM) simulations. Each sample encodes building geometry, release location, wind speed, and time as multi-channel input images, with the corresponding gas dispersion concentration field is recorded as the output. Two cGAN architectures, Image-to-Image Translation (Pix2Pix) and its high-resolution variant (Pix2PixHD), are employed to learn the mapping from input features to dispersion fields. Model performance is evaluated using four complementary metrics: Fraction within a Factor of Two (FAC2) for prediction accuracy, Normalized Root Mean Square Error (NRMSE) for precision, Fractional Bias (FB) for systematic error, and Structural Similarity Index (SSIM) for spatial pattern fidelity. A case study is conducted across a 1176 km^2^ urban district in China. The results demonstrate that under varying wind speeds (0.5–1.5 m/s) and temporal scales (5–60 s), and across five morphological categories, the Pix2PixHD-based model achieves 92.5% prediction accuracy and reproduces 97.6% of the spatial patterns. The proposed framework accelerates computation by approximately 18,000 times compared to traditional CFD, reducing inference time to under 0.1 s per scenario. This sub-second capability enables real-time concentration field estimation for emergency management, and provides a physically informed, computationally feasible forward model that can potentially support sensor-based gas source localization and detection network planning in complex urban environments.

## 1. Introduction

Industrial chemical spills, accidental hazardous gas releases, and even deliberate dispersals pose severe threats to public health and safety in densely built urban areas [1,2]. In the critical first few minutes following such an incident, rapidly locating the emission source and delineating the contaminated zone are essential for guiding evacuation and initiating emergency response. Urban building clusters fundamentally alter pollutant transport: flow blockage, street-canyon channeling, and wake recirculation generate highly asymmetric, non-Gaussian concentration fields within the urban canopy. These complex flow regimes are closely linked to local building density and morphological configuration, making the spatial structure of the dispersion field strongly site-dependent. Consequently, the fidelity of the forward dispersion model directly governs the accuracy of source localization algorithms; an oversimplified model inevitably introduces systematic bias into posterior source estimates.

Achieving such rapid and accurate dispersion prediction in complex urban settings remains a significant challenge. Mobile robotic sensing systems have shown promise for gas source localization through in situ concentration measurements [3], yet their performance is fundamentally constrained by the accuracy of the underlying dispersion model used for likelihood estimation in source inference frameworks. Sensor placement optimization strategies [4] further highlight the critical dependence of detection efficiency on reliable gas dispersion modeling in complex environments. Wind tunnel studies [5] offer controlled validation but face scalability challenges. Gaussian plume models, widely adopted in source localization due to their computational efficiency [6], cannot adequately capture building-induced flow modifications, leading to inaccurate likelihood functions for Bayesian source inference in urban settings. Although Computational Fluid Dynamics (CFD) approaches, including Large Eddy Simulation (LES), Reynolds-Averaged Navier–Stokes (RANS) methods, and the Lattice Boltzmann Method (LBM), can resolve building obstruction effects and deliver high-fidelity simulations [7,8,9], their computational demands remain prohibitive for real-time applications, typically requiring hours to days per simulation scenario. In summary, these established methodologies have yet to achieve the necessary balance between physical accuracy and computational efficiency required for emergency response operations.

Recent advances in computational and data-driven approaches have significantly improved atmospheric dispersion modeling. Gas dispersion simulators have been developed to support mobile robot olfaction and sensor validation in realistic environments [10], while multi-graph models capture spatial correlations between monitoring stations [11], and sequence-to-sequence architectures utilize upwind sensor data to enhance prediction accuracy [12]. Physics-informed deep learning has also shown promise in reconstructing complex flow fields from sparse measurements [13], further motivating data-driven approaches to dispersion modeling. More recently, image-based machine learning methods have demonstrated the ability to reconstruct gas concentration distributions from sparse sensor observations with higher accuracy than conventional spatial interpolation [14]. While these sensor-driven approaches provide valuable tools for post-event concentration estimation, source localization frameworks require a fundamentally different ability: a forward model that can rapidly generate full concentration fields from hypothesized source parameters (location, release rate, meteorological conditions) without requiring prior sensor observations. This fundamental asymmetry between sensor-driven estimation and forward prediction motivates the development of a dedicated generative surrogate. Such a forward model must be evaluated repeatedly within iterative Bayesian inference loops, placing stringent demands on both physical fidelity and computational speed. This requirement points toward image-based generative models that are capable of producing continuous, high-resolution dispersion fields directly from source and environmental conditions.

Conditional Generative Adversarial Networks (cGANs) provide a transformative approach to dispersion modeling by utilizing image-to-image translation to generate hazard gas dispersion fields. This technique has demonstrated considerable success across multiple domains: in indoor environments, cGANs have achieved rapid predictions of complex airflow and hazard gas dispersion patterns [15,16]; in fire scenarios, they have yielded substantial computational acceleration while maintaining high predictive accuracy [17,18]; and at the urban scales, cGANs have been effectively applied to wind field prediction, reducing computation time from hours, as is needed using conventional CFD simulations, to seconds without compromising fidelity [19,20,21]. Furthermore, insights from high-fidelity CFD studies have critically highlighted the influence of urban morphology on gas dispersion patterns [22]. Nevertheless, the application of cGANs to coupled advection–diffusion processes, particularly for predicting hazard gas dispersion in realistic urban settings with complex building obstructions, remains an underexplored area of research. A key challenge in this direction is maintaining prediction fidelity across diverse urban configurations, as building layouts vary substantially between districts, giving rise to fundamentally different flow and dispersion regimes that a single model may fail to capture. Given the established relationship between urban wind fields and gas dispersion patterns [23], along with the demonstrated effectiveness of cGANs for rapid wind field modeling, extending cGAN frameworks with morphology-aware learning strategies offers a promising avenue for generating accurate dispersion predictions that generalize across heterogeneous urban environments, thereby improving emergency response preparedness and enabling reliable real-time risk assessment. It is also worth noting that such real-time surrogate models hold potential as computationally efficient forward models to support sensor-based source localization and detection network planning in complex urban environments when conventional CFD is too slow and Gaussian plume models lack the fidelity needed to resolve building obstruction effects.

This study proposes a morphology-guided conditional generative adversarial network (cGAN) framework for the rapid prediction of hazard gas dispersion field in urban environment, explicitly accounting for building obstruction effects. The proposed approach effectively bridges the gap between computational efficiency and high-resolution concentration field generation required for emergency response. By integrating urban morphology analysis with generative modeling, the framework achieves morphology-specific learning and delivers sub-second inference speeds. The remainder of this paper is structured as follows: the methodology is detailed in Section 2, including morphology clustering, LBM simulations, and cGAN architecture; the case study and dataset preparation are introduced in Section 3; model performance under different wind conditions, temporal evolution, and urban morphologies is evaluated in Section 4; and key findings are summarized along with future research directions in Section 5.

## 2. Methodology

**Figure 1:**
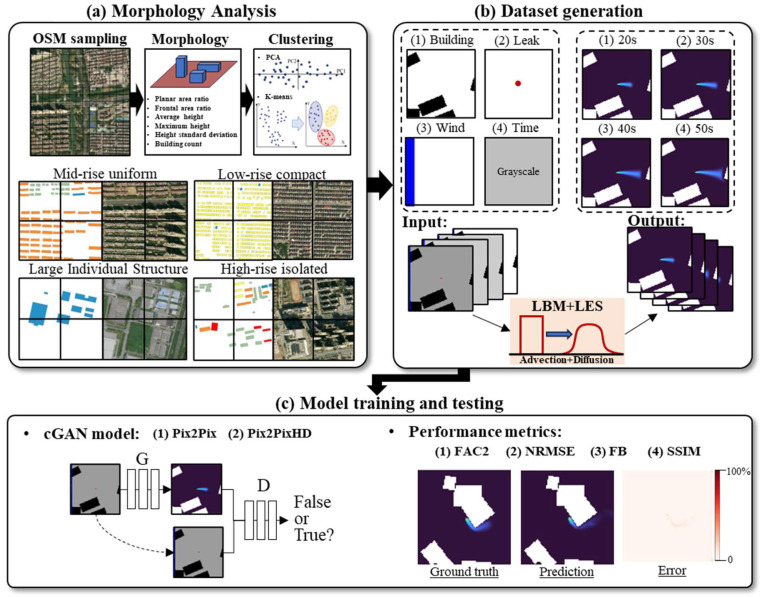
Workflow of the proposed surrogate cGAN framework for rapid prediction of hazard gas dispersion fields in urban environments: (**a**) urban morphology analysis based on OSM sampling, morphology descriptors, and PCA/K-means clustering, where points in the PCA plot represent grid cells and grouped points indicate clustered morphology classes; representative examples of typical urban morphology types are shown below; (**b**) dataset generation, where black regions denote buildings, the blue bar denotes the inflow wind input, the red dot denotes the gas source, grayscale denotes elapsed time, and colored output frames denote gas dispersion fields at different times; (**c**) cGAN models, including Pix2Pix and Pix2PixHD and performance evaluation using FAC2, NRMSE, FB, and SSIM.

The proposed framework employs three components to facilitate the rapid prediction of the hazard gas dispersion field, as illustrated in Figure 1: (a) urban morphology is classified by clustering six geometric parameters characterizing building configuration; (b) high-fidelity training datasets are generated via LBM simulations for each morphology-classified urban cell type, with each sample pairing multi-channel inputs (building geometry from the classified morphological category, gas source location, wind speed/direction, and elapsed time) with the corresponding LBM-simulated gas dispersion field as the ground truth output; and (c) cGAN-based surrogate models are developed using Image-to-Image Translation (Pix2Pix) and its high-resolution variant (Pix2PixHD) architectures to predict gas dispersion fields. Four metrics are used to evaluate the method’s performance: the Fraction within a Factor of Two (FAC2) for accuracy, the Normalized Root Mean Square Error (NRMSE) for precision, the Fractional Bias (FB) for systematic error, and the Structural Similarity Index (SSIM) for spatial pattern quality. These components are elaborated in Section 2.1, Section 2.2 and Section 2.3 respectively, and the evaluation metrics are detailed in Section 2.4.

### 2.1. Urban Morphology Analysis and Clustering

**Figure 2:**
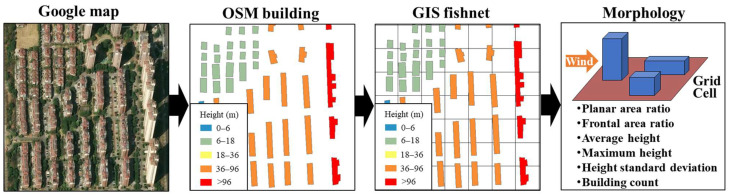
GIS fishnet sampling of building footprints extracted from OSM. Building footprints extracted from OSM are shown in black, and the GIS fishnet grid cells are overlaid on the study area.

Hazard gas dispersion in urban environments is fundamentally influenced by building obstruction effects, requiring the systematic transformation of irregular urban layouts into quantifiable geometric patterns. As illustrated in Figure 2, the methodology consists of four main steps. First, the area of concern is identified using satellite imagery. Second, building footprints and heights are extracted from the OpenStreetMap (OSM) database, which offers comprehensive three-dimensional geometric coverage across the district. Third, a Geographic Information System (GIS) fishnet sampling approach discretizes the continuous urban landscape into regular 50 × 50 m grid cells, converting heterogeneous building distributions into standardized spatial units. Finally, six key morphological parameters are computed for each grid cell.

The 50 × 50 m grid size is consistent with the characteristic size of urban blocks, making each cell representative of a distinct micro-environment for hazard gas dispersion modeling. This scale captures neighborhood-level flow interactions while maintaining computational tractability. Within each cell, the three-dimensional building configuration is quantified using parameters known to influence dispersion mechanisms. Based on established wind tunnel and CFD studies [22,24,25,26], six morphological parameters are selected to collectively represent the aerodynamic properties governing dispersion patterns, as summarized in Table 1.

The mathematical definitions of these parameters are as follows. The maximum building height $H_{\max}$ represents the tallest structure within the grid cell:

(1) $$H_{\max}=\max\{H_{i}|i\in B\}$$

where $H_{i}$ is the height of *i*-th building; *B* denotes the set of all buildings within the grid.

The area-weighted mean height $H_{\mathrm{ave}}$ provides a representative measure of the overall building volume:

(2) $$H_{\mathrm{ave}}=\frac{\sum \left(A_{Pi}\times H_{i}\right)}{\sum A_{Pi}}$$

where $A_{Pi}$ represent the planar area; $H_{i}$ is the height of *i*-th building in the grid cell.

The height standard deviation $\sigma_{H}$ quantifies the variability of building heights:

(3) $$\sigma_{H}=\sqrt{\frac{\sum A_{Pi}{\left(H_{\mathrm{ave}}-H_{i}\right)}^{2}}{\sum A_{Pi}\times {H_{\mathrm{ave}}}^{2}}}$$

The building count $N_{b}$ is the total number of buildings within the grid cell:

(4) $$N_{b}=\mathrm{count}(B)$$

The planar area ratio $\lambda_{P}$ is defined as the ratio of the total building footprint area to grid cell area:

(5) $$\lambda_{P}=\frac{\sum A_{Pi}}{A_{grid}}$$

where $A_{grid}$ is the area of the grid cell, which is 2500 m^2^.

The frontal area ratio $\lambda_{F}$ represents the ratio of the total building frontal area to the grid cell cross-sectional area:

(6) $$\lambda_{F}=\frac{\sum A_{Fi}}{A_{grid}}$$

where $A_{Fi}$ is the frontal area of *i*-th building in the grid cell.

These morphological parameters exhibit inherent correlations due to urban planning constraints. Principal Component Analysis (PCA) is applied to transform these correlated features into orthogonal components. The first 2–3 principal components typically capture over 90% of the total variance, effectively reducing dimensionality while preserving essential morphological information.

K-means clustering is then applied to the PCA-transformed features to categorize the urban landscape into distinct morphological types. The optimal number of clusters *k* is determined using validation metrics: the Silhouette coefficient, which measures cluster cohesion and separation; the Calinski–Harabasz index (C-H index), which quantifies between-cluster and within-cluster variance. The resulting morphological clusters provide a structured sampling framework for training data generation, ensuring a balanced representation of various urban configurations in the dataset.

### 2.2. Physics-Based Simulation for a High-Fidelity Dataset

The quality of the training dataset fundamentally determines the performance of the surrogate cGAN model, necessitating high-fidelity simulations that accurately capture the complex physics of hazard gas dispersion in an urban environment. The Lattice Boltzmann Method with Large Eddy Simulation (LBM-LES) [27,28] was selected as the numerical framework, primarily due to its recognized computational efficiency for simulating turbulent flows at urban scales compared to traditional CFD methods, while maintaining satisfactory accuracy for the intended application. While three-dimensional simulations provide a more comprehensive representation of flow physics, the two-dimensional LBM approach is well-suited for modeling ground-level hazard gas releases. This suitability arises because horizontal dispersion is the primary governing mechanism for near-surface concentration patterns, which are critical for assessing human exposure risk. Furthermore, the incorporation of key building geometric parameters allows the model to effectively represent the influence of urban canopy on local flow dynamics. However, the present two-dimensional formulation is intended to approximate near-surface concentration evolution rather than fully resolve three-dimensional wake structures such as downwash, vertical recirculation, and plumes rising around tall, isolated buildings. These effects are only partially reflected through the morphology descriptors, especially maximum building height, mean height, height variability, and frontal area ratio, but are not explicitly resolved in the current model. The D2Q9 lattice model shows the evolution of particle distribution functions through streaming and collision processes:

(7) $$f_{i}(x+e_{i}\Delta t,t+\Delta t)=f_{i}(x,t)+\Omega_{i}(x,t)$$

where $f_{i}$ represents the distribution function in the *i*-th direction; $e_{i}$ denotes the discrete velocity vectors; and $\Omega_{i}$ is the collision operator.

**Figure 3:**
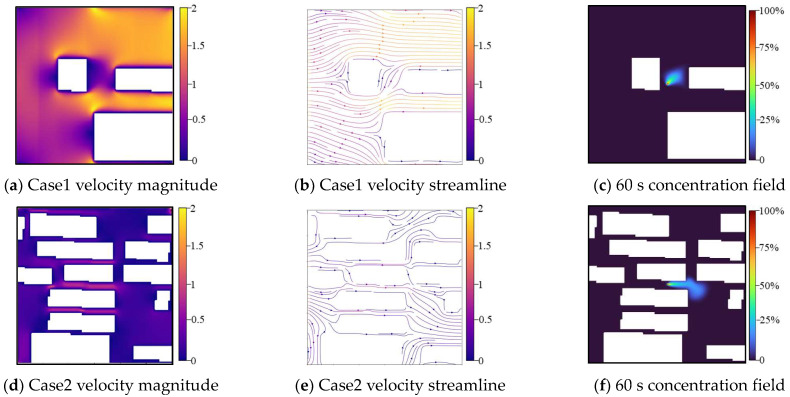
Velocity and gas dispersion fields of LBM-LES results.

The Multiple-Relaxation-Time (MRT) collision operator is employed to enhance numerical stability via moment space transformation, whereas the Smagorinsky subgrid model (*Cs* = 0.1) captures turbulent diffusion, which is critical for achieving accurate urban dispersion simulations. As illustrated in Figure 3, the simulation results depict complex flow patterns through velocity magnitude contours and streamline visualizations, along with gas dispersion concentration fields that illustrate the deformation and dispersion processes of hazard gas plumes. The model outputs the temporal evolution of concentration in a 60 s period, sampled at 5 s intervals, yielding 12 sequential snapshots for analysis.

**Figure 4:**
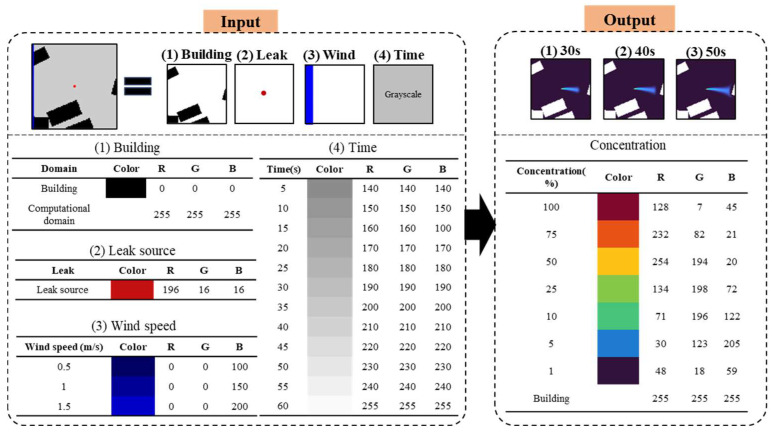
Paired input–output image encoding scheme used for training. In the input image, black regions indicate building geometries, the red dot indicates the leak source location, blue regions indicate the wind-speed bar, and grayscale squares indicate the elapsed time. In the output image, colors represent gas concentration levels, with warmer colors indicating higher concentrations and cooler colors indicating lower concentrations.

These simulated physical fields, containing two-dimensional velocity vectors and scalar concentration values at each grid point, must be encoded into paired image sets to train the cGAN model. As shown in Figure 4, the input image integrates four data layers: (1) building geometries, represented in black RGB (0,0,0) against a white background RGB (255,255,255); (2) a leak source marked by a single red pixel RGB (196,16,16); (3) a wind speed bar, with blue intensity scaling linearly from RGB (0,0,100) at 0.5 m/s to RGB(0,0,200) at 1.5 m/s; and (4) a time indicator using grayscale values ranging from RGB (140,140,140) at 5 s to RGB (255,255,255) at 60 s. The corresponding output image represents the gas dispersion field using the Turbo colormap [20], with high concentrations shown in deep red RGB (128,7,45), medium values in yellow RGB (254,194,20), and low concentrations in blue RGB (48,18,59). Building areas are masked in white throughout.

To enhance the generalization ability of the model, data augmentation is performed through a vertical flipping of the spatial domain, encompassing both building layouts and gas dispersion fields. This geometric transformation generates mirrored urban configurations while maintaining the original wind direction, thereby preserving physical consistency in all synthesized samples. The augmented dataset comprising these physically realistic variations effectively expands the diversity of training instances and improves the model’s robustness across different urban morphological patterns.

### 2.3. Conditional Generative Adversarial Network (cGAN) Architecture

**Figure 5:**
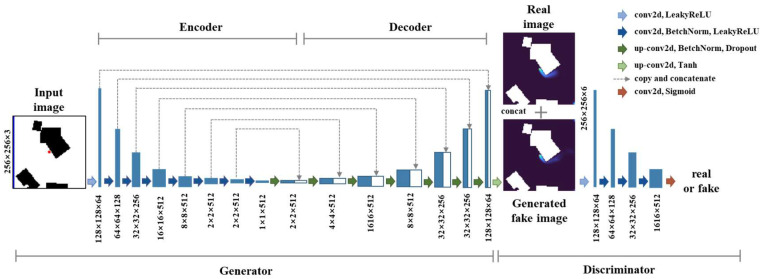
Architecture of the Pix2Pix model with the U-Net generator and PatchGAN discriminator.

Figure 5 illustrates the baseline Pix2Pix architecture, which consists of a U-Net generator and a PatchGAN discriminator. The generator incorporates symmetric encoder–decoder pathways for hierarchical feature extraction. The encoder progressively downsamples input images from 256 × 256 resolution through a series of convolutional layers (yielding feature maps at 128 × 128, 64 × 64, down to 1 × 1 resolution), thereby capturing hierarchical features ranging from local building edges to global spatial patterns. The decoder reconstructs gas dispersion fields via transposed convolutions, gradually upsampling feature representations from the bottleneck layer back to the original 256 × 256 resolution. Critical to this process, skip connections directly propagate multi-scale features from each encoder layer to the corresponding decoder layer, mitigating information loss and preserving the high-frequency details essential for resolving sharp concentration gradients near building structures. The PatchGAN discriminator operates on local image patches (70 × 70 pixels) rather than full images, efficiently enforcing local perceptual realism.

Although Pix2Pix delivers satisfactory performance at 256 × 256 resolution, capturing finer spatial details necessitates higher-resolution modeling. Pix2PixHD addresses this limitation through a multi-scale generation framework: a global generator first establishes the overall plume structure at a lower resolution, while a local enhancer network subsequently refines the details to achieve 512 × 512 resolution outputs. Three distinct discriminators operate at different scales to ensure consistency across resolution levels. Additional loss terms, including feature matching loss and perceptual loss based on pre-trained Visual Geometry Group (VGG) networks, are incorporated to enhance structural fidelity beyond pixel-level accuracy, thereby preserving critical flow structures and the concentration gradients necessary for assessing exposure risks.

### 2.4. Evaluation Performance Metrics

**Figure 6:**
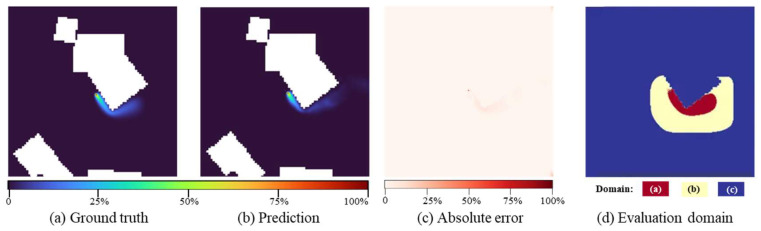
Illustration for model performance evaluation.

Model performance is evaluated using four metrics following established atmospheric dispersion modeling standards [29]: Fraction of predictions within a Factor of Two (FAC2), Fractional Bias (FB), Normalized Root Mean Square Error (NRMSE), and Structural Similarity Index (SSIM). To provide a focused and physically relevant assessment, the evaluation specifically targets contaminated regions where prediction accuracy critically influences emergency decision-making. This approach prevents metric distortion caused by inclusion of zero-concentration areas. As illustrated in Figure 6, while the predicted and ground-truth concentration fields show broad visual agreement, the absolute error map reveals localized discrepancies, highlighting the necessity of a carefully constrained evaluation domain. The evaluation domain D is formally defined as

(8) $$D=D_{\mathrm{core}}\cup D_{\mathrm{buffer}}$$

where $D_{\mathrm{core}}=\{(x,y)|C_{g}(x,y)\geq 0.01\times C_{\max}\}$ represents the core contamination zone, indicated by the red domain (a) in Figure 6d; $D_{\mathrm{buffer}}$ denotes a 5 m surrounding buffer shown as the yellow domain (b). The blue region (c) corresponds to excluded areas, which include building footprints and uncontaminated zones. For each test scenario, all pixels within D are indexed as *i* = 1, 2, …, *N*, where *N* denotes the total number of evaluation points capturing the concentration gradients essential for determining evacuation boundaries.

#### 2.4.1. Factor of Two (FAC2)

The Factor of Two (FAC2) quantifies the proportion of predictions within a factor of two of ground truth, providing a robust measure of model accuracy:

(9) $$\mathrm{FAC}2=\frac{1}{N}\sum_{i=1}^{N}I(0.5\leq \frac{C_{\mathrm{pred},i}}{C_{\mathrm{gt},i}}\leq 2.0)$$

where *N* is the total number of evaluation points within D; I(⋅) is the indicator function returning 1 when the condition is satisfied and 0 otherwise; $C_{\mathrm{pred},i}$ and $C_{\mathrm{gt},i}$ denote the predicted and ground truth concentrations at point *i* of the field. FAC2 values range from 0 to 1, with higher values indicating better prediction accuracy.

#### 2.4.2. Fractional Bias (FB)

The Fractional Bias (FB) measures systematic over-prediction and under-prediction tendencies through normalized mean differences:

(10) $$\mathrm{FB}=\frac{2(\bar{C_{\mathrm{gt}}}-\bar{C_{\mathrm{pred}}})}{\bar{C_{\mathrm{gt}}}+\bar{C_{\mathrm{pred}}}}$$

(11) $$\bar{C_{\mathrm{gt}}}=\frac{1}{N}\sum_{i=1}^{N}C_{\mathrm{gt},i}$$

(12) $$\bar{C_{\mathrm{pred}}}=\frac{1}{N}\sum_{i=1}^{N}C_{\mathrm{pred},i}$$

where $\bar{C_{\mathrm{pred}}}$ and $\bar{C_{\mathrm{gt}}}$ represent the mean predicted and ground truth concentrations over the evaluation domain D. FB ranges from −2 to 2, with negative values indicating over-prediction and positive values indicating under-prediction.

#### 2.4.3. Normalized Root Mean Square Error (NRMSE)

The Normalized Root Mean Square Error (NRMSE) quantifies the overall magnitude of prediction errors normalized by the ground truth concentration range:

(13) $$\mathrm{NRMSE}=\frac{\sqrt{\frac{1}{N}\sum_{i=1}^{N}{\left(C_{\mathrm{pred},i}-C_{\mathrm{gt},i}\right)}^{2}}}{\max(C_{\mathrm{gt}})-\min(C_{\mathrm{gt}})}$$

This range-based normalization enables comparison across different scenarios while maintaining a dimensionless metric. NRMSE values range from 0 to +∞, with lower values indicating better performance.

#### 2.4.4. Structural Similarity Index (SSIM)

The Structural Similarity Index (SSIM) evaluates the preservation of spatial patterns through a joint assessment of luminance, contrast, and structural correlation:

(14) $$\mathrm{SSIM}=\frac{\left(2\bar{C_{\mathrm{gt}}}\bar{C_{\mathrm{pred}}}+c_{1})(2\sigma_{\mathrm{gt},\mathrm{pred}}+c_{2}\right)}{\left({\bar{C_{\mathrm{gt}}}}^{2}+{\bar{C_{\mathrm{pred}}}}^{2}+c_{1})(\sigma_{\mathrm{gt}}^{2}+\sigma_{\mathrm{pred}}^{2}+c_{2}\right)}$$

(15) $$\sigma_{\mathrm{gt}}^{2}=\frac{1}{N-1}\sum_{i=1}^{N}(C_{\mathrm{gt},i}-\bar{C_{gt}}{)}^{2}$$

(16) $$\sigma_{\mathrm{pred}}^{2}=\frac{1}{N-1}\sum_{i=1}^{N}(C_{\mathrm{pred},i}-\bar{C_{\mathrm{pred}}}{)}^{2}$$

(17) $$\sigma_{\mathrm{gt},\mathrm{pred}}=\frac{1}{N-1}\sum_{i=1}^{N}\left(C_{\mathrm{pred},i}-\bar{C_{\mathrm{pred}}})(C_{\mathrm{gt},i}-\bar{C_{\mathrm{gt}}}\right)$$

where $\sigma_{\mathrm{gt}}^{2}$ is the local variances in ground truth; $\sigma_{\mathrm{pred}}^{2}$ is local variances in prediction; $\sigma_{\mathrm{gt},\mathrm{pred}}$ is local covariance between ground truth and predicted fields; and $c_{1}={\left(0.01L\right)}^{2}$, $c_{2}={\left(0.03L\right)}^{2}$ are stabilization constants, with *L* representing the concentration dynamic range, $L=\max(C_{\mathrm{gt},i})-\min(C_{\mathrm{gt},i})$. SSIM values range from 0 to 1, with values exceeding 0.9 indicating excellent structural preservation.

## 3. Dataset and Experimental Setup

### 3.1. Study Area and Data Preparation

**Figure 7:**
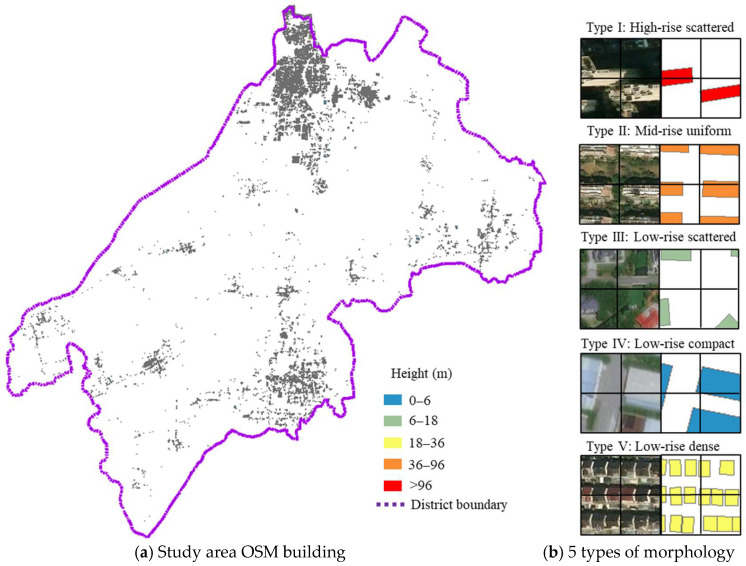
Morphology analysis in the study area.

A representative urban district spanning 1176 km^2^ in China was selected as a demonstration project due to its varied building morphologies, which range from traditional low-rise neighborhoods to modern high-rise developments, as shown in Figure 7. The area encompasses mixed land uses, including residential, commercial, and industrial zones, offering a comprehensive representation of typical urban configurations in Chinese cities.

Building vector data for the study area was obtained from OpenStreetMap (OSM). A Geographic Information System (GIS) fishnet sampling approach was then applied to discretize the area into grid cells with a dimension of 50 × 50 m. After filtering cells containing negligible built environment (primarily open spaces, water bodies, and agricultural land), 28,859 cells with buildings were retained for analysis. Six morphological parameters were computed for each grid: maximum height ranges from 3 m to 262 m, planar area ratio varies from 0.05 to 0.99, and building count spans 1 to 36 structures per cell. PCA reduced these parameters to three components explaining 91.7% of the variance.

**Figure 8:**
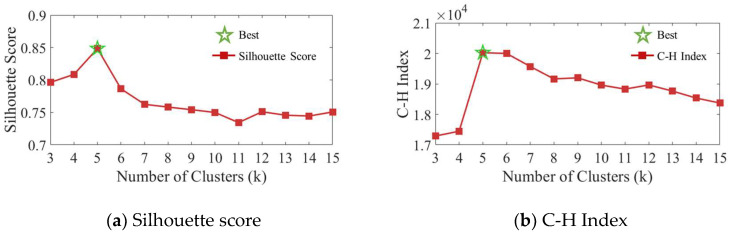
Clustering quality metrics for determining the optimal number of morphological types. The red line with square markers represents the Silhouette score, and the green star markers represent the Calinski–Harabasz index.

The optimal number of clusters is determined through systematic evaluation of clustering quality metrics across *k* = 3 to 15. As illustrated in Figure 8, the Silhouette Score peaks at *k* = 5, with a value of 0.85, while the Calinski–Harabasz Index (C-H Index) continues increasing beyond *k* = 5, reaching approximately 2.0 × 10^4^. The final clustering yields five morphology types ranging from high-rise isolated to low-rise dense configurations, with low-rise isolated dominating at 58% of the study area.

The morphological characteristics of the five identified clusters, summarized in Table 2, reveal distinct urban patterns. Type I, high-rise isolated, exhibits extreme vertical development ($H_{\max}$ = 261.88 m, $H_{\mathrm{ave}}$ = 89.36 m) with a small planar area ratio ($\lambda_{P}$ = 0.22), characteristic of a central business district. Type II, mid-rise uniform, shows balanced parameters ($H_{\max}$ = 92.70 m, $\lambda_{P}$ = 0.40) typical of modern residential compounds. Type III, low-rise isolated, dominates 58% of the area and features sparse development ($H_{\max}$ = 21.63 m, $\lambda_{P}$ = 0.20) representing suburban and transitional zones. Type IV, low-rise compact, demonstrates an extensive planar area ratio ($\lambda_{P}$ = 0.81) despite low heights, and is common in industrial areas. Type V, low-rise dense, combines a high number of buildings ($N_{b}$ = 18.18) with a moderate planar area ratio, reflecting traditional neighborhoods where numerous small structures create complex flow patterns.

### 3.2. Simulation Configuration

LBM-LES simulations generate training data at 0.1m spatial resolution with domain size 500 × 500 pixels representing 50 × 50 m areas. Wind speeds of 0.5, 1.0, and 1.5 m/s were selected to represent typical urban pedestrian-level wind. As low wind speeds reduce ventilation, hazard gas accumulation and exposure risks increase, making low-wind-speed scenarios essential for emergency response planning. A uniform velocity inlet condition is applied at the upstream boundary, while free boundary conditions are imposed at the downstream and lateral boundaries. No-slip boundary conditions are enforced on building surfaces and the ground plane. These boundary settings should be interpreted as a near-ground idealization for morphology-conditioned horizontal dispersion, rather than a full representation of vertically evolving urban flow fields. Simulations first establish fully developed turbulent flow fields and then introduce hazard gas sources and track dispersion for 60 s with outputs at 5 s intervals.

Each simulation requires approximately 25 min of computational time on a standard Intel i7 CPU. The total dataset comprises 550 training scenarios and 50 test scenarios after vertical flip augmentation, ensuring a minimum of 50 training and 5 test cases per morphological cluster to prevent model bias. This balanced allocation strategy, rather than proportional sampling based on real-world distribution, prevents model bias toward the dominant Type III category (57.96%) and ensures the adequate representation of minority classes such as Type I (3.76%).

### 3.3. Model Training Configuration

Both cGAN architectures are implemented on NVIDIA RTX 4090 GPUs with identical optimization settings: Adam (β_1_ = 0.5, β_2_ = 0.999), learning rate 0.002, batch size 1. As shown in Table 3, Pix2Pix trains for 100 epochs (12 h) at 256 × 256 resolution with standard losses (λ_adv_ = 1.0, λ_L1_ = 100.0). The Pix2PixHD-based model achieves 512 × 512 resolution through staged training: 50 epochs for the global generator and 50 epochs for joint optimization (48 h total), with additional feature matching and perceptual losses (λ_FM_ = 10.0, λ_VGG_ = 10.0) to preserve fine-scale structures.

**Figure 9:**
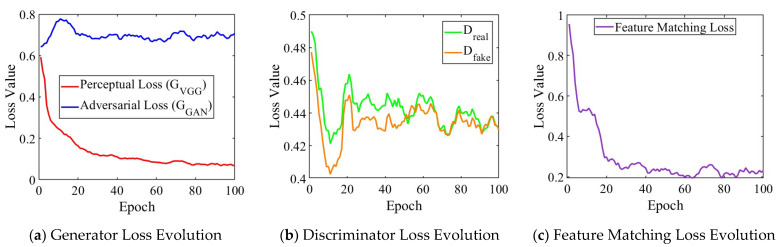
Training loss evolution of Pix2PixHD-based model.

The training convergence of Pix2PixHD-based model remains stable throughout the optimization process, as shown in Figure 9. The perceptual loss decreases from 0.6 to 0.1 over 100 epochs, indicating improved structural similarity, while adversarial loss remains within a stable range around 0.7, maintaining generation quality without mode collapse. The feature matching loss also exhibits a clear downward trend, especially during the early training stage, indicating progressively improved alignment between generated and real samples in the discriminator feature space. Discriminator losses for real and fake samples converge to 0.44, confirming a balanced adversarial training where neither network dominates. This equilibrium emerges after 20 epochs and persists throughout training, validating the staged approach for high-resolution generation. Despite the four-fold resolution increase, inference time only increases from 0.07 s (Pix2Pix-based) to 0.08 s (Pix2PixHD-based), with both meeting real-time requirements, showing about an 18,000-fold speedup compared to the physics-based simulation.

## 4. Results and Discussion

### 4.1. Overall Prediction Performance

A comprehensive performance evaluation across the test dataset, presented in Table 4, demonstrates that the Pix2PixHD-based model consistently outperforms the Pix2Pix-based model across all metrics. The FAC2 metric improves from 0.897 (Pix2Pix-based model) to 0.925 (Pix2PixHD-based model), indicating enhanced reliability in concentration predictions. The negative FB for the Pix2PixHD-based model (-0.114) indicates a slight overprediction, providing conservative estimates that are beneficial for emergency response planning. In contrast, the positive FB for Pix2Pix (0.118) suggests underprediction, which could compromise safety margins. The reduction in NRMSE from 0.026 to 0.015 represents a 42% improvement in quantitative accuracy. Furthermore, the SSIM approaching unity (0.976) confirms that the Pix2PixHD-based model preserves plume morphology and concentration gradients, which are essential for evacuation planning.

**Figure 10:**
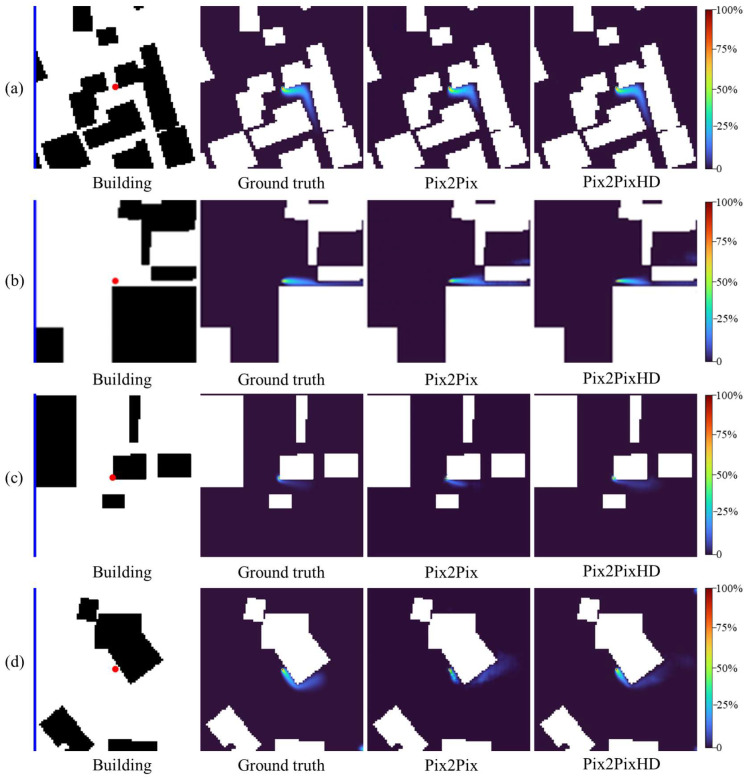
Comparison of 60 s gas dispersion fields for four representative cases: (**a**) Case 1; (**b**) Case 2; (**c**) Case 3; and (**d**) Case 4. In each case, from left to right, the panels show the input image (building layout in black, inflow wind bar in blue, and source location as a red dot), the ground-truth concentration field, the prediction of the Pix2Pix-based model, and the prediction of the Pix2PixHD-based model. The color bar indicates the gas concentration level.

The visual comparison of the 60 s gas dispersion fields across four test cases in Figure 10 reveals that while both models predict the general dispersion pattern, Pix2PixHD-based model demonstrates superior performance in reproducing fine-scale features and maintaining gas dispersion field fidelity.

### 4.2. Sensitivity Analysis

#### 4.2.1. Wind Speed Sensitivity Analysis

The performance evaluation across different wind speeds, as detailed in Table 5, shows systematic variations in model accuracy. Both models achieve optimal performance at low wind speed (0.5 m/s), with the Pix2PixHD-based model reaching FAC2 = 0.941, outperforming the Pix2Pix-based model. Performance gradually decreases with increasing wind speed, as FAC2 drops to 0.911 at 1.5 m/s for the Pix2PixHD-based model and 0.874 for Pix2Pix. This trend reflects the increasing challenge of capturing enhanced advection effects at higher wind speeds. The Pix2PixHD-based model maintains stable performance across all wind speeds (NRMSE = 0.015, FB ≈ −0.1, SSIM > 0.97), while Pix2Pix shows degrading accuracy with increasing velocity (FB from 0.037 to 0.200). The consistent FAC2 > 0.9 for the Pix2PixHD-based model validates the model’s robustness under varying wind conditions.

#### 4.2.2. Temporal Evolution Analysis

**Figure 11:**
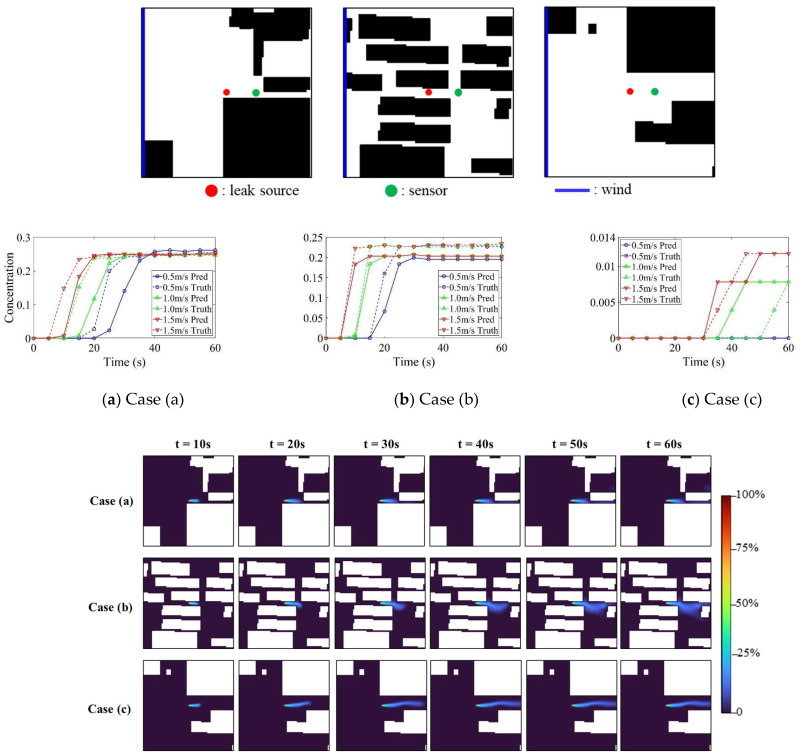
Temporal concentration evolution for three cases.

Prediction accuracy evolves systematically in the 60 s simulation period, as shown in Table 6 and Figure 11. Early-stage predictions achieve the highest accuracy, with performance gradually declining as dispersion complexity increases. The FAC2 metric decreases monotonically from 0.956 at 5s to 0.902 at 60s for the Pix2PixHD-based model, which consistently outperforms the Pix2Pix-based model throughout the temporal evolution. Despite this decline, all values remain well above acceptability thresholds.

A notable observation in Table 6 is the contrasting FB behavior between the two models. For the Pix2Pix-based model, FB exhibits a sign reversal from −0.262 at t = 5 s to +0.376 at t = 10 s, followed by persistent oscillations throughout the simulation period. This instability arises because, at the earliest stage, the concentration field is compact and spatially concentrated, causing the model to slightly overpredict. As the plume rapidly expands beyond 10 s, the evolving concentration gradients exceed the representational capacity of the 256 × 256 resolution, resulting in systematic underprediction with temporal fluctuations. In contrast, the Pix2PixHD-based model exhibits a monotonic and smooth FB transition from −0.358 at 5 s to −0.027 at 60 s, confirming that the higher-resolution architecture provides substantially more stable modeling of temporal dispersion evolution.

Sensor-level validation for three cases, illustrated in Figure 11, confirms the proposed model’s ability to accurately capture temporal concentration evolution. The downstream sensor data demonstrate that the model effectively tracks both the concentration rise and stabilization phases.

#### 4.2.3. Morphology-Specific Performance

Analysis of model performance across different urban morphologies, summarized in Table 7, reveals systematic variations in prediction accuracy. Type III (low-rise isolated) achieves the highest accuracy due to the minimal building obstruction and relatively simple flow patterns, whereas Type V (low-rise dense) exhibits the lowest accuracy, reflecting the challenges in capturing complex wake interactions from numerous closely spaced structures. Types I, II, and IV show comparable performance despite their distinct geometric characteristics.

Type V (low-rise dense) exhibits the lowest accuracy (FAC2 = 0.891; SSIM = 0.962) with the highest prediction bias (FB = -0.18), indicating the difficulty in modeling complex flow interactions within dense building arrangements. The 4.5% FAC2 degradation from the best- to worst-performing morphology aligns with the known limitations of data-driven approaches in predicting building obstruction effects. Nevertheless, all morphology types maintain FAC2 > 0.89 and SSIM > 0.95, confirming the model’s robust generalization capability without catastrophic failure in complex urban environments.

### 4.3. Comparison with Existing Approaches

To contextualize the performance of the proposed framework, Table 8 provides a qualitative comparison with established dispersion modeling approaches and recent cGAN-based surrogate models.

Gaussian plume models offer real-time computational speed but fundamentally cannot resolve building-induced flow modifications, leading to substantial prediction errors in complex urban settings. Conventional CFD and LBM methods deliver high-fidelity results but require approximately 25 min per scenario, rendering them impractical for real-time emergency response. The proposed framework bridges this gap by simultaneously achieving sub-second inference (0.08 s), high predictive accuracy (FAC2 = 0.925), and the ability to resolve building obstruction effects on gas dispersion fields across diverse urban morphologies. This combination of speed, fidelity, and morphological generalization distinguishes the proposed approach from existing methods and enables its deployment as a computationally feasible forward model for real-time emergency management applications.

## 5. Conclusions

This study presents a morphology-guided conditional generative adversarial network framework for the rapid prediction of hazard gas dispersion in urban environment. The urban area is discretized into grid cells characterized by six morphological parameters and classified into distinct categories via k-means clustering. High-fidelity dispersion datasets, generated via the Lattice Boltzmann Method (LBM) simulations, are employed as training data. Each sample encodes building geometry, release location, wind speed, and temporal factors as inputs, with corresponding gas dispersion fields as outputs. The framework employs Pix2Pix and Pix2PixHD architectures as cGAN-based surrogate models to map the input features. Model performance is evaluated using multiple metrics: the Fraction within a Factor of Two (FAC2) for predictive accuracy, the Normalized Root Mean Square Error (NRMSE) for precision, the Fractional Bias (FB) for systematic error assessment, and the Structural Similarity Index (SSIM) for spatial pattern fidelity. The proposed framework effectively transforms computationally intensive CFD simulations into sub-second predictive tasks, while maintaining high accuracy, thus offering substantial potential for real-time emergency response applications. The specific conclusions drawn from this study are as follows:

(1) The proposed framework, utilizing the Pix2PixHD architecture, achieves high predictive accuracy, with an FAC2 of 0.925 and SSIM of 0.976 on the complete test dataset, demonstrating robustness across varying wind speeds, temporal evolution, and diverse urban morphological configurations.
(2) The framework attains a computational speedup of 18,000 times compared to physics-based simulations, reducing processing time from 25 min per scenario using LBM-LES to 0.08 s with Pix2PixHD.
(3) The Pix2PixHD-based model exhibits significant improvements over the baseline Pix2Pix-based model, achieving a 42% reduction in NRMSE (from 0.026 to 0.015) while maintaining conservative predictions (FB = −0.114) that are advantageous for safety-critical applications.

This study focuses on single point-source releases with continuous ground-level emissions and employs two-dimensional gas dispersion fields, which may not fully capture vertical plume rise effects. Additionally, steady-state wind conditions are assumed without considering atmospheric stability variations or transient meteorological changes that influence real-world dispersion. Accordingly, the proposed framework is directly applicable to ground-level, single point-source continuous releases under steady-state wind conditions (0.5–1.5 m/s) in urban areas with building heights up to approximately 260 m. Extension to elevated releases, multi-source scenarios, time-varying meteorological conditions, and three-dimensional dispersion fields requires further investigation. Therefore, the present framework is most directly applicable to short-term emergency scenarios in which local wind conditions can be approximated as quasi-steady over the prediction window. For rapidly changing wind direction or wind speed, future work will incorporate time-varying meteorological forcing into the multi-channel input representation, for example, by introducing sequential wind-condition channels or short-term wind-history features. Future validation against representative three-dimensional CFD/LBM simulations and/or wind-tunnel measurements will be carried out, particularly for high-rise isolated morphologies, to quantify the influence of vertical wake effects on near-ground concentration prediction.

Overall, this study demonstrates that cGAN can serve as practical and efficient alternatives to computationally prohibitive CFD simulations for hazard gas dispersion in urban environment, successfully balancing the requirements of speed, accuracy, and gas dispersion field generation necessary for emergency response. Future work will also explore integrating the proposed surrogate model as a forward-dispersion engine within Bayesian source localization frameworks and sensor placement optimization, leveraging its sub-second inference speed to enable real-time probabilistic inversion from sparse sensor observations.

## Figures and Tables

**Table 1:** Six morphological parameters and their influence on urban hazard gas dispersion.

Parameters	Symbol	Physical Significance	Reference
Maximum building height	$H_{\max}$	Vertical mixing extent, downwash potential	[22,26]
Area-weighted mean height	$H_{\mathrm{ave}}$	Overall volume displacement, boundary layer depth	[22,26]
Height standard deviation	$\sigma_{H}$	Turbulence generation, flow channeling	[25]
Building count	$N_{b}$	Flow obstacle density, wake interactions	[24]
Planar area ratio	$\lambda_{P}$	Horizontal blockage, ground-level ventilation	[22,24,25]
Frontal area ratio	$\lambda_{F}$	Wind resistance, pressure gradient formation	[26]

**Table 2:** Characteristics of identified urban morphology clusters.

Category	Type I: High-Rise Scattered	Type II: Mid-Rise Uniform	Type III: Low-Rise Isolated	Type IV: Low-Rise Compact	Type V: Low-Rise Dense
$\lambda_{F}$	1.74	0.86	0.17	0.42	0.37
$\lambda_{P}$	0.22	0.40	0.20	0.81	0.61
$H_{\max}$ (m)	261.88	92.70	21.63	26.55	27.90
$H_{\mathrm{ave}}$ (m)	89.36	40.58	20.14	24.21	23.29
$\sigma_{H}$	0.00180	0.00456	0.00190	0.00128	0.00317
$N_{b}$	3.17	5.37	3.44	4.47	18.18
Number	1085	4135	16726	4650	2263
Percentage	3.76%	14.33%	57.96%	16.11%	7.84%

**Table 3:** Training configuration of Pix2Pix and Pix2PixHD models.

Model	Pix2Pix	Pix2PixHD
Input size	256 × 256	512 × 512 (256 × 256)
Output size	256 × 256	512 × 512
Epoch	100	100
Batch_size	1	1
Learning rate	0.002	0.002
Training information	Random Normal Distribution Initialization, Adam	Random Normal Distribution Initialization, Adam
Training duration	12 H	48 H
Prediction duration	0.07 s	0.08 s

**Table 4:** Comparative performance metrics between Pix2Pix-based and Pix2PixHD-based models.

Model	FAC2	FB	NRMSE	SSIM
Pix2Pix	0.897	0.118	0.026	0.958
Pix2PixHD	0.925	−0.114	0.015	0.976

**Table 5:** Performance metrics across different wind speeds.

Model	Pix2Pix-Based				Pix2PixHD-Based			
Wind Speed (m/s)	FAC2	FB	NRMSE	SSIM	FAC2	FB	NRMSE	SSIM
0.5	0.919	0.037	0.027	0.963	0.941	−0.131	0.015	0.983
1	0.897	0.117	0.026	0.958	0.924	−0.103	0.015	0.975
1.5	0.874	0.200	0.026	0.952	0.911	−0.092	0.015	0.971
Summation	0.897	0.118	0.026	0.958	0.925	−0.114	0.015	0.976

**Table 6:** Temporal evolution of performance metrics for Pix2Pix and Pix2PixHD.

Model	Pix2Pix-Based				Pix2PixHD-Based			
Time(s)	FAC2	FB	NRMSE	SSIM	FAC2	FB	NRMSE	SSIM
5	0.945	−0.262	0.023	0.976	0.956	−0.358	0.013	0.992
10	0.942	0.376	0.021	0.976	0.951	−0.235	0.014	0.988
15	0.928	0.312	0.021	0.971	0.943	−0.130	0.015	0.984
20	0.919	0.140	0.022	0.967	0.938	−0.157	0.015	0.980
25	0.911	0.312	0.022	0.965	0.932	−0.125	0.016	0.978
30	0.901	0.181	0.021	0.961	0.926	−0.092	0.016	0.975
35	0.890	0.049	0.021	0.958	0.918	−0.074	0.016	0.972
40	0.884	0.050	0.022	0.956	0.915	−0.076	0.016	0.972
45	0.880	0.152	0.022	0.956	0.912	−0.065	0.016	0.970
50	0.874	0.146	0.022	0.954	0.908	−0.052	0.016	0.969
55	0.865	0.148	0.023	0.953	0.903	−0.028	0.016	0.969
60	0.861	0.200	0.023	0.952	0.902	−0.027	0.016	0.969

**Table 7:** Performance metrics of the Pix2PixHD-based model across urban morphology types.

Type	I	II	III	IV	V
FAC2	0.922	0.918	0.932	0.913	0.891
SSIM	0.961	0.975	0.977	0.970	0.952
NRMSE	0.013	0.016	0.014	0.016	0.018
FB	−0.13	−0.11	−0.10	−0.13	−0.18
Type	High-rise isolated	Mid-rise uniform	Low-rise isolated	Low-rise compact	Low-rise dense

**Table 8:** Comparison of the proposed framework with existing dispersion modeling approaches.

Method	Application	Inference Time	Accuracy	Building Effects
Gaussian plume	Urban source localization	Real-time	Low	Cannot resolve
CFD	Urban dispersion	~25 min per scenario	High	Fully resolved
Pix2PixHD	Urban gas dispersion	0.08 s	FAC2 = 0.925, SSIM = 0.976	Fully resolved

## Data Availability

The data presented in this study are available on request from the corresponding author.
